# 

**Published:** 1828-04

**Authors:** 


DISTRICT OF OHIO, *Sct.*

Be it remembered, that on the first day of March, in the year of our Lord, one thousand eight hundred and twenty-eight, and in the fifty-first year of American Independence, Daniel Drake, of said district, hath deposited in this office the title of a book, the right whereof he claims as proprietor, in the words following, to wit:

"The Western Journal of the Medical and Physical Sciences. Edited by Daniel Drake, M. D. Late Professor of Theory and Practice of Medicine in Transylvania University: Surgeon of the Cincinnati Eye Infirmary: Member of the Philadelphia Academy of Natural Science: of the American Philosophical Society: and of the Royal Wernerian Society of Natural History, Edinburgh—"

In conformity to the act of the Congress of the United States, entitled "An act for the encouragement of learning, by securing the copies of Maps, Charts and Books to the proprietors of such copies, during the time therein mentioned;" and also of the act entitled "An act supplementary to an act for the encouragement of learning, by securing the copies of Maps, Charts and Books to the Authors and proprietors of such copies, during the time therein mentioned, and extending the benefits thereof to the arts of designing, engraving, and etching historical and other prints."

WILLIAM KEY BOND,
*Clerk of the District of Ohio.*

### Necrology.

**DIED,** on the 18th of March, at the place of his nativity, in Mason County, Kentucky, of a chronic disease of the larnyx, complicated with a malady of the heart, Dr. JOHN DRAKE, aged 24 years. The deceased was a graduate of the Transylvania School, but studied the elements of the profession in this City. His strong native talents had been cultivated by intense and successful application; and with elevated professional aspirations, he associated the purest views of moral excellence. His numerous friends, both in and out of the profession, will regret to learn his premature death.

